# Introduction to the Special Issue: Advances in Metacognition, Learning, and Reactivity

**DOI:** 10.3390/jintelligence13040046

**Published:** 2025-04-09

**Authors:** Chunliang Yang, Liang Luo

**Affiliations:** 1 Institute of Developmental Psychology, Faculty of Psychology, Beijing Normal University, Beijing 100875, China; 2 Beijing Key Laboratory of Applied Experimental Psychology, National Demonstration Center for Experimental Psychology Education, Beijing Normal University, Beijing 100875, China; 3 State Key Laboratory of Cognitive Neuroscience and Learning, Beijing Normal University, Beijing 100875, China

Metacognition, particularly the ability to monitor and regulate cognitive processes, plays a crucial role in effective learning. Metacognitive monitoring allows learners to assess their knowledge mastery status and subjectively evaluate the likelihood of remembering the content of studied materials in the future. One of the most extensively studied forms of metacognitive judgment is judgments of learning (JOLs), where individuals predict the likelihood of remembering a studied item in a later memory test (Koriat 1997; Thiede and Dunlosky 1999). JOLs are crucial for guiding learners’ study strategy selection and effective allocation of cognitive resources. Typically, learners regulate their study behaviors (e.g., when, what, and how to study) based on their JOLs (Finn 2008; Metcalfe 2009; Thiede et al. 2003; Yang et al. 2017). However, learners frequently hold flawed mental models of how learning and memory function, resulting in inaccurate metacognitive assessments and ineffective management of their own learning (Bjork et al. 2013; Kornell and Bjork 2009). For instance, learners tend to prefer studying category exemplars in blocks and erroneously believe that blocked learning is more beneficial for category induction than interleaved learning, even though interleaved learning is actually more effective (Li et al. 2025; Sun et al. 2022). This underscores the need to understand why learners frequently misjudge their knowledge mastery level, identify potential factors affecting metacognitive monitoring accuracy, and develop effective interventions to promote the accuracy of self-assessments (Rhodes and Tauber 2011; Yang et al. 2021b).

Recent studies have documented that making JOLs can also reactively alter (typically enhance) memory in a direct way, a phenomenon referred to as the reactivity effect (Li et al. 2023; Zhao et al. 2022). The reactivity effect suggests that JOLs are not merely passive measures of metamemory monitoring but can actively shape memory performance by directing attention, reallocating cognitive resources, or altering the perceived importance of the material being studied. Understanding the mechanisms underlying JOL reactivity and its impacts on learning is critical for improving educational instruction.

Each paper in this Special Issue provides valuable insights into the potential mechanisms, influencing factors, and interventions aimed at enhancing the accuracy of metacognitive monitoring. Several papers further contribute to the understanding of the influencing factors and potential mechanisms underlying the JOL reactivity effect, as well as the impact of JOLs on learning educational materials.

We briefly introduce the important contributions of each paper, starting with those in the area of mechanisms and influencing factors of metacognitive monitoring. Zhou and Jia (2023) found that item difficulty affects confidence judgments through the intermediate variable, that is, processing fluency. Their study provided evidence for cue integration from the perspective of the cue utilization theory (Hertzog et al. 2013; Koriat 1997), demonstrating that individuals use both intrinsic cues and mnemonic cues to construct confidence judgments. From the perspective of mindfulness, Yin et al. (2023) demonstrated that dispositional mindfulness is positively related to the relative accuracy of JOLs, while test anxiety weakens the positive effect of mindfulness on JOL accuracy. Another study by Sun and Jiang (2023) observed that age-related differences in emotional regulation influences metacognitive monitoring, with older adults potentially being more susceptible to metacognitive illusions that skew their learning judgments in a positive direction than younger adults.

Another prominent theme focuses on interventions for improving learners’ beliefs about the relative effectiveness of different learning strategies. Previous studies found that learners do not always appreciate the mnemonic benefits of effective learning strategies, such as practice testing, over restudying (Hui et al. 2022; Toppino et al. 2018). Yet, research has repeatedly confirmed that retrieval practice improves learning more than restudying does (Yang et al. 2018; Yang et al. 2021a). These metacognitive illusions associated with effective study strategies lead to underemployment of these effective strategies during self-regulated learning. Hence, studies presented in this Special Issue explored various interventions to improve learners’ beliefs about effective study strategies. For instance, Rivers (2023) examined how different instructional approaches—test experience, direct instruction, and their combination—can enhance learners’ beliefs about the effectiveness of practice testing in promoting learning. The results showed that both test experience and direct instruction independently helped students form more accurate beliefs about the power of practice testing in enhancing learning. However, the combination of test experience and direct instruction is no more effective than either technique alone. Such findings underscore that experiencing the effectiveness of practice testing on one’s own memory performance and receiving instructions about its benefits may be critical components of interventions designed to improve students’ beliefs about and self-regulated use of practice testing. Wang et al. (2023b) found that combining prompts and feedback in a hypermedia environment helps students better monitor their learning process, improves the absolute accuracy of their meta-comprehension judgments, and ultimately results in better learning outcomes. In short, both studies provided evidence that some intervention techniques are available to improve learners’ metacognitive beliefs and accuracy.

In addition, this Special Issue includes studies on other aspects of metacognition, such as the critical role of metacognition in creative thinking (Jiang et al. 2023) and scientific literacy (Xie et al. 2023). For instance, Jiang et al. (2023) utilized behavioral tasks and eye tracking techniques to investigate how individuals with higher metacognitive ability perform better on tasks requiring both divergent and convergent thinking. Divergent thinking involves generating creative, varied ideas, while convergent thinking focuses on finding the best solution to a given problem. The results showed that individuals with high metacognitive ability are better at creative thinking, especially in terms of divergent thinking. Additionally, individuals with high metacognitive ability exhibit greater fixation and saccade count, as well as smaller average saccade amplitude on task stimuli, reflecting that they are better at focusing attention on key information and monitoring and regulating cognition in a top-down manner. These findings also suggest that metacognitive ability not only enhances the creative process but also improves individuals’ capacity to regulate and adapt their thinking strategies. Meanwhile, Xie et al. (2023) explored the influence path of metacognitive reading strategies (metacognitive understanding and remembering strategies, metacognitive summarizing strategies, and metacognitive assessing credibility strategies) on scientific literacy based on a database including 11,420 15-year-old students from four Chinese provinces (Beijing, Shanghai, Jiangsu, and Zhejiang) who took part in the Programme for International Student Assessment (PISA) in 2018. Their findings indicate that metacognitive assessing credibility strategies have the strongest positive impact on scientific literacy, with notable gender differences in the effects of the three metacognitive reading strategies.

On the other hand, some of the submissions in this Special Issue, like the study of Rivers et al. (2023), focus on the reasons why making JOLs reactively enhances memory performance. Rivers et al. investigated whether attentional reorienting during encoding contributes to the positive reactivity effect on memory for related word pairs. Their findings showed that positive reactivity for related word pairs cannot be solely explained by attentional reorienting during encoding. They proposed that the cue-strengthening hypothesis may be one possible explanation, which asserts that if the cue used to inform JOLs is relevant to a criterion test, positive reactivity will occur for that material because participants often use the relatedness of two words in a pair to form JOLs. Witherby et al. (2023) further examined the cue-strengthening theory by using interactive imagery, instructing participants to create a relationship between the cue and target words by forming a mental image of the two words interacting. However, their outcomes are inconsistent with this theory, indicating that associative relationships created by forming interactive mental images do not appear to be strengthened by making JOLs. In contrast, the results of Double (2023) support the central idea of the cue processing account for reactivity, namely that JOLs prompt participants to process salient cues to decide whether or not to utilize them when making JOLs. Another explanation of the JOL reactivity effect is the item-specific processing hypothesis, which posits that item-level JOLs enhance later memory performance by promoting item-specific processing. The study by Chang and Brainerd (2024) challenges the item-specific processing account. They argue that whether JOLs predominantly engage participants with item-specific or relational processing depends on the interaction between learning stimuli and JOLs.

Several contributions in this Special Issue explore the reactivity effect of JOLs on the learning of complex materials, contributing to a deeper understanding of how metacognitive strategies interact with task complexity in learning processes. Specifically, Wang et al. (2023a) provided empirical evidence that delayed JOLs can promote subsequent performance in learning of new categories whereas the promoting effect is observed only for materials of medium or high difficulty. Hausman and Kubik (2023) showed that delayed JOLs did not improve text comprehension after one week. Furthermore, Zhao et al. (2023) demonstrated that making JOLs following retrieval practice had no reactivity effect on the learning of educationally related texts. In addition to the aforementioned studies, this Special Issue includes articles on the cue sources and cue utilization patterns involved in social mentalizing during two-person interactions (Dai et al. 2023). It also addresses factors influencing memory, such as the impact of expectations on subsequent memory retrieval of emotional words (Xiao and Nie 2023).

In conclusion, the studies included in this Special Issue profitably increase our understanding of the mechanisms underlying metacognitive monitoring, and bring new excitement to these fields. These issues are directly relevant to factors influencing metacognitive judgments, interventions aimed at promoting monitoring accuracy, and self-use of effective study strategies during self-regulated learning. We hope that this Special Issue encourages further research with educational materials, in classroom settings, and focusing on developing effective tools to eliminate or reduce reactivity in future metacognition research and the practical use of reactivity in learning settings. Understanding the mechanisms underlying the reactivity effect of metacognitive judgments remains critical and challenging.
